# The Asymmetric Spillover Effects of Retirement on Disability: Evidence From China

**DOI:** 10.1093/geroni/igae074

**Published:** 2024-08-23

**Authors:** Anying Bai, Zhuang Hao, Huihui Cheng, Simiao Chen, Yu Jiang

**Affiliations:** School of Population Medicine and Public Health, Chinese Academy of Medical Sciences and Peking Union Medical College, Beijing, China; Nuffield Department of Medicine, University of Oxford, Oxford, UK; School of Economics and Management, Beihang University, Beijing, China; Laboratory for Low-Carbon Intelligent Governance, Beihang University, Beijing, China; Rutgers Business School, Rutgers University, New Brunswick, New Jersey, USA; School of Population Medicine and Public Health, Chinese Academy of Medical Sciences and Peking Union Medical College, Beijing, China; Faculty of Medicine and University Hospital, Heidelberg Institute of Global Health (HIGH), Heidelberg University, Heidelberg, Germany; School of Population Medicine and Public Health, Chinese Academy of Medical Sciences and Peking Union Medical College, Beijing, China; School of Health Policy and Management, Chinese Academy of Medical Sciences and Peking Union Medical College, Beijing, China

**Keywords:** ADL/IADL disability status, Heterogeneity analysis, Nonparametric, Regression discontinuity design, mechanism

## Abstract

**Background and Objectives:**

Recent research has explored the spillover effects of retirement on spousal well-being, yet limited attention has been given to the short-term impact on spousal disability. This study explored the asymmetric spillover impact of retirement on spouses’ disability severity among a national cohort of urban residents in China.

**Research Design and Methods:**

Utilizing 4 waves of data (2011–2018) from the China Health and Retirement Longitudinal Survey, we employ a nonparametric regression discontinuity design to estimate the short-term effect of retirement on spousal disability severity. Disability is assessed based on their ability to perform activities of daily living (ADLs) and instrumental activities of daily living (IADLs). Furthermore, we conduct heterogeneity analysis stratified by factors such as the husband’s retirement status, health conditions, lifestyle behaviors, and the wife’s educational level. Additionally, we explore potential mechanisms including changes in health behaviors, emotions, and disease diagnoses.

**Results:**

Our findings indicate that wives’ retirement has a significant favorable short-term effect on husbands’ ADL scores, with a magnitude of −0.644 points (−9.78% relative to baseline). A significant beneficial effect of wives’ retirement on the prevalence of husbands’ difficulty in dressing, bathing, and eating was observed with substantial magnitudes of 0.075, 0.201, and 0.051 points, respectively. Various heterogeneity analyses and sensitivity tests confirmed the robustness of our results. The positive spillover effect of wives’ retirement likely results from reduced negative emotions in husbands. In contrast, husbands’ retirement does not affect the prevalence of ADL/IADL disability in their wives.

**Discussion and Implications:**

Underscoring the gender asymmetry in the effects of spousal retirement on disability, this study emphasizes the need for tailored policies considering men’s and women’s distinct disability experiences.

**Translational Significance:** This study highlights gender differences in the short-term impact of spousal retirement on disability within the context of Chinese urban residents. Wives’ retirement reduces husbands’ disability rates, but husbands’ retirement does not affect wives. Wives’ retirement positively affects husbands’ health, likely by reducing negative emotions. It emphasizes the importance of gender-specific approaches in healthcare and policy, showcasing the potential benefits of wives’ retirement for husbands’ independence. Additionally, it underscores the need for tailored support systems addressing the unique challenges women face in maintaining functional abilities during retirement.

In developing countries, people aged 60 years and older represent a fast-growing population. Maintaining the well-being of this demographic, particularly retirees, has become a major concern for policy-makers (Na & Streim, 2017). Retirement, a life-changing event, initiates a cascade of consequences for the family members of retirees. These include re-negotiating household responsibilities and the loss of a regular source of income, potentially influencing family members’ health behaviors and health status through cross-spousal spillover effects (Coe & Zamarro, 2011).

In recent years, a small but quickly growing body of literature has focused on the cross-spousal effects of retirement on health. Retirement may trigger role loss (changes in income and work-related social networks), which may create pressure on family members (Adams et al., 2002). Empiric studies examining the health impacts of spousal retirement have also emerged. However, the majority of past research has focused on the spousal spillover effect of women’s retirement on their husbands’ health behaviors (Tobias Müller & Mujaheed Shaikh, 2018; Wang & Shultz, 2010; Zang, 2020) and mental health (Bertoni & Brunello, 2017; Johnson & Hall, 1988; Szinovacz, 2000; Zang, 2020), yielding inconsistent results. One previous research from China proposed that a husband’s retirement has a deteriorating effect on his wife’s health, primarily due to lifestyle changes and increased psychological pressure (Wang & Shultz, 2010). Post-retirement adjustments may also increase the demands retired husbands place on their wives to perform homemaking tasks, leading to greater mental strain (Johnson & Hall, 1988; Szinovacz, 2000). Another study in Japan found that a husband’s retirement deteriorates his wife’s mental health by increasing her stress level, causing depression and reducing her ability to sleep, and negatively affects her subjective health status (Bertoni & Brunello, 2017). Conversely, one study in China indicated that a husband’s retirement improves his wife’s physical and mental well-being through enhanced social interactions and exercise (Zang, 2020). Another study using European data further observed that although an individual’s retirement boosts their spouse’s physical activity, it also increases smoking and alcohol consumption (Tobias Müller & Mujaheed Shaikh, 2018). Besides examining the spillover effect of husbands’ retirement, researchers have explored the impact of wives’ retirement on their husbands. Nonetheless, findings from these studies have presented a mixed picture. Certain investigations in both China (Chen et al., 2021) and Australia (Atalay & Zhu, 2018) have noted a favorable impact on the mental well-being of husbands when their wives retire, whereas contrasting findings have indicated no substantial correlation between a wife’s retirement and her husband’s self-rated health, reported chronic conditions, or mental health among Chinese population (Chen, 2022). These divergent outcomes underscore potential gender and social context-related variations in the retirement-induced spillover effects on spousal health-related outcomes, emphasizing the necessity for a meticulous, case-specific approach to the study of these effects (Chen et al., 2020). Such mixed results also imply systematic differences between developing countries and the developed economies typically examined in prior research concerning the direction and magnitude of retirement externality.

Amid growing attention to the spillover impacts of retirement on health, there remains a significant gap: No study has yet explored the cross-spousal effects of retirement on disability and its severity. Older individuals experiencing disability require physical assistance due to degenerated body functions (Jette, 2006; Vaughan et al., 2016). Disability affects the quality of life and is associated with institutionalization, increased healthcare costs, and mortality risk (Ferrucci et al., 1997). A study across 54 countries estimated the global prevalence of disability among the older population at 39% (Mitra & Sambamoorthi, 2014), with projections indicating a rise in disabled Chinese older adults from 102 million in 2020 to 138 million in 2030 (Gong et al., 2022). Disability measures encompass the ability to perform standard activities of daily living (ADLs) and instrumental activities of daily living (IADLs). ADLs are fundamental activities for independent living at home (e.g., bathing, feeding oneself, etc.), and IADLs are more complex activities that require a higher level of autonomy and cognitive function and are necessary for independent life in the community (Vaughan et al., 2016). Because disability among older adults is common (Jette, 2021) and China is one of the world’s fastest-aging countries (Man et al., 2021), the impending retirement of cohorts approaching the retirement age could pose considerable challenges due to the increasing costs of service related to disability. Understanding the factors that contribute to disability can help policy-makers better support older adults and avert financial hardship. Many epidemiological studies have identified major cardiovascular disease risk factors are also risk factors for disability in older age. These include obesity (Hubert et al., 1993; Pinsky et al., 1985; Reed et al., 1998), smoking (d’Orsi et al., 2014; Pinsky et al., 1987), elevated blood pressure (Hubert et al., 1993; Pinsky et al., 1985; Reed et al., 1998), diabetes mellitus (Gregg et al., 2000; Miller et al., 2004), and depressive symptoms (Barry et al., 2009; Wang et al., 2002). Spouses and other family members frequently provide informal care to disabled individuals. However, the impact of this care on spousal disability, particularly in relation to diverse cardiovascular disease risk factor statuses, remains understudied. Therefore, understanding how retirement and spousal retirement affect disability in both the overall population and various subgroups can inform policies related to caregiving and family leave.

The impact of spousal retirement on disability could be manifested through three potential pathways: changing health behaviors (H1), reducing negative emotions (H2), and increasing the diagnosis rate of chronic diseases (H3). To begin with, upon retirement, both men and women are freed from the demands of earning a living, and their health behaviors may change consequently. Health-related behaviors are highly correlated between spouses (Fletcher & Marksteiner, 2017; Meyler et al., 2007), and the theory of concordance emphasizes that wives often attempt to control their spouses’ behaviors to keep them healthy (Umberson, 1992). For example, married individuals tend to engage less frequently in risky behaviors such as not wearing seatbelts, drinking outside the home, and eating irregular, low-quality meals than their unmarried counterparts (Schone & Weinick, 1998; Umberson, 1987). Healthier post-retirement lifestyles compared to pre-retirement lifestyles, for example, with regard to physical activity (Henkens et al., 2008) and social engagement (Gao et al., 2018; Zunzunegui et al., 2005), are associated with a lower prevalence of ADL disability. By contrast, unhealthy behaviors, such as the onset of smoking, have been shown to increase incident functional impairment (Hajek & König, 2016), and alcohol consumption may affect Chinese older adults’ ability to carry out ADLs (Lee et al., 2021). Second, negative emotions (e.g., anger, anxiety, and depression) can seriously damage one’s health and play an important role in the development of a variety of diseases (Carnethon et al., 2003). Theories of emotional contagion suggest that individuals living in interdependent relationships with a partner mutually experience affective or emotional states (Goodman & Shippy, 2002). Depressive symptoms correlate positively with ADL disability among the older population (Deng & Paul, 2018; Kaneko et al., 2007). Finally, spousal retirement might increase the diagnosis rate of chronic diseases among husbands. Spousal retirement may lead to increased utilization of healthcare services, including preventative care and disease management, which can result in earlier diagnosis and treatment of chronic diseases. Because most chronic conditions are known as precursors for developing disability, early screening and identification of illnesses might help prevent the occurrence and development of these secondary consequences (Grimby et al., 1988; Verbrugge & Jette, 1994; W. W. Hung et al., 2012).

To address the knowledge gap regarding the causal effects of spousal retirement, we used a rigorous quasi-experimental approach, the regression discontinuity design (RDD), to empirically explore the asymmetric spillover impact of retirement on spouses’ disability severity among a national cohort of urban residents in China. Moreover, we analyzed potential mechanisms that may link spousal retirement to disability, including its effects on health behaviors, emotions, and disease diagnosis.

## Method

### Data Source and Study Population

We used data from the 2011, 2013, 2015, and 2018 waves of the China Health and Retirement Longitudinal Study (CHARLS), a nationally representative longitudinal survey of the middle-aged and elderly population (aged 45 years and above) and their spouses in China. The CHARLS survey was derived from a multistage-stratified probability-proportional-to-size sampling, ensuring a baseline sample closely matched the demographic distribution captured in the 2010 Chinese census. A total of 17,705 respondents were interviewed during the baseline survey in 2011, and respondents were followed up every 2 years through face-to-face, computer-assisted personal interviews. The original CHARLS study was approved by the Ethical Review Committee of Peking University (IRB00001052–11015). The original CHARLS study was approved by the Ethical Review Committee of Peking University (IRB00001052–11015). All participants signed informed consent at the time of initial participation. Further details on the CHARLS survey design are available elsewhere (Zhao et al., 2014).

We pooled the four waves of CHARLS and used the interview year and household identification number to link husbands and wives. We excluded 16,954 individuals who either lacked birth-year information or were interviewed in 2012, 2014, or 2016, resulting in a sample of 60,279 individuals. The sample was then restricted to urban residents aged between 40 and 75 (Zhang et al., 2018), reducing the sample size to 11,372 individuals. Further exclusions were made for the self-employed, those who did not process retirement, and individuals who neither worked nor reported “processed retirement,” further reducing the sample to 9,442 observations. “Processed retirement” means that an employee reaching the statutory retirement age (SRA) leaves the current job after going through all the formalities with the employer and local government. Finally, observations with missing information on retirement status, age, gender, or educational background were excluded, leaving us with a sample of 7,227 individual-year observations. Supplementary Figure 1 provides additional details on the study population inclusion process, with nonresponse analysis results comparing included and excluded populations available in Supplementary Table 1. We attempted to retrieve 103 subjects interviewed in 2012, 2014, and 2016 with complete data and found that the primary conclusions remained unchanged. Thus, the change in the number of individuals was not substantial, and we chose not to amend the study sample to incorporate these 103 individuals.

### Retirement Definition

China’s statutory retirement policy, officially launched in 1978, stipulates an SRA of 60 for men and 50 for women in urban China (55 for female civil servants, who only constitute a small portion of the population; Lei & Liu, 2018; Li et al., 2016). This policy does not vary by province and is enforced strictly in the public sector. In the private sector, employees do not have to retire but become eligible for a pension at the SRA (Zhang et al., 2018). Although a “progressive delay retirement scheme” has recently been proposed to raise the retirement ages gradually, the policy is still under debate (Zhang et al., 2018).

Retirement status in the CHARLS questionnaire was determined based on responses to the following questions: (1) “Have you completed retirement procedures (including early retirement) or internal retirement?” and (2) “Work includes all kinds of labor excluding doing your own housework, whether you earn wages or not. Are you sure that you didn’t work for at least three months during your lifetime?” We classified respondents as retired if they met the following three conditions: (1) self-reported that they had retired; (2) were working prior to reported retirement, had not quit the labor force before retirement, and did not work after processed retirement (because we conceptualize “retirement” as a change from working to non-working status); and (3) were older than 45 years (because people who “retire” very early are likely to have stopped working for reasons other than normal age-related retirement).

### Outcome Variables

Disability status was determined based on participants’ responses regarding difficulties in carrying out ADLs/IADLs. A disability index (ADL/IADL score) measures the occurrence and severity of disability. The ADL index used in CHARLS is derived from the Katz index (Katz et al., 1963), encompassing dressing, bathing, eating, walking across the room, using the toilet, and controlling urination or defecation. The IADL index used in CHARLS is based on Lawton’s index (Lawton & Brody, 1969) and includes five items: managing money, taking medication, shopping, meal preparation, and doing housework. There were four levels of possible answers to each question about the ability to perform a given ADL/IADL task: “no difficulty,” “have difficulty but can still do it,” “have difficulty and need help,” and “cannot do it.” For each item in the ADL and IADL indices, a dichotomous variable with an assigned value of one if a respondent had difficulty with or could not perform it and zero otherwise. A dependence score ranging from one (total independence) to four (total dependence) was simultaneously awarded, and cumulative ADL/IADL scores were calculated as the sum of the respondent’s scores for each item to measure the overall dependence level of each respondent. Cumulative ADL scores ranged from 6 to 24, and cumulative IADL scores ranged from 5 to 20, with a higher score indicating a greater level of dependence with respect to performing daily activities (Li et al., 2022).

### Statistical Analysis

We adopt a fuzzy RDD as our identification strategy (Chen et al., 2021; Ebeid & Oguzoglu, 2023; Hao et al., 2024). This approach could be applied when treatment is determined by whether a continuous “assignment variable” exceeds an exogenously determined threshold. Specifically, we exploited the exogenous changes in spousal retirement likelihood caused by China’s SRA (60 for men and 50 for women) among urban residents to identify the causal effect of spousal retirement on disability severity and specific disability indicators.

RDD estimates the causal effect of retirement without active randomization by exploiting the fact that people with an age just above and just below the SRA threshold are likely exchangeable with each other, except for their retirement likelihood. We were thus able to estimate the causal effect of spousal retirement by contrasting individuals in close proximity to the threshold regarding their disability severity. To achieve this, we employed a local linear regression discontinuity estimation technique utilizing a triangular kernel, along with bias-corrected confidence intervals following previous studies (Calonico et al., 2014a, 2014b, 2017, 2019a; Hao et al., 2024; Zang, 2020; Zhang et al., 2018). Bandwidth selection is performed utilizing the mean square error (MSE)-optimal bandwidth selectors (Calonico et al., 2014a, 2019a). Moreover, to address potential within-individual error term correlations, standard errors were clustered at the individual level, consistent with the recommendations of previous research (Eibich & Siedler, 2020; Zhang et al., 2018).

**Figure 1.**
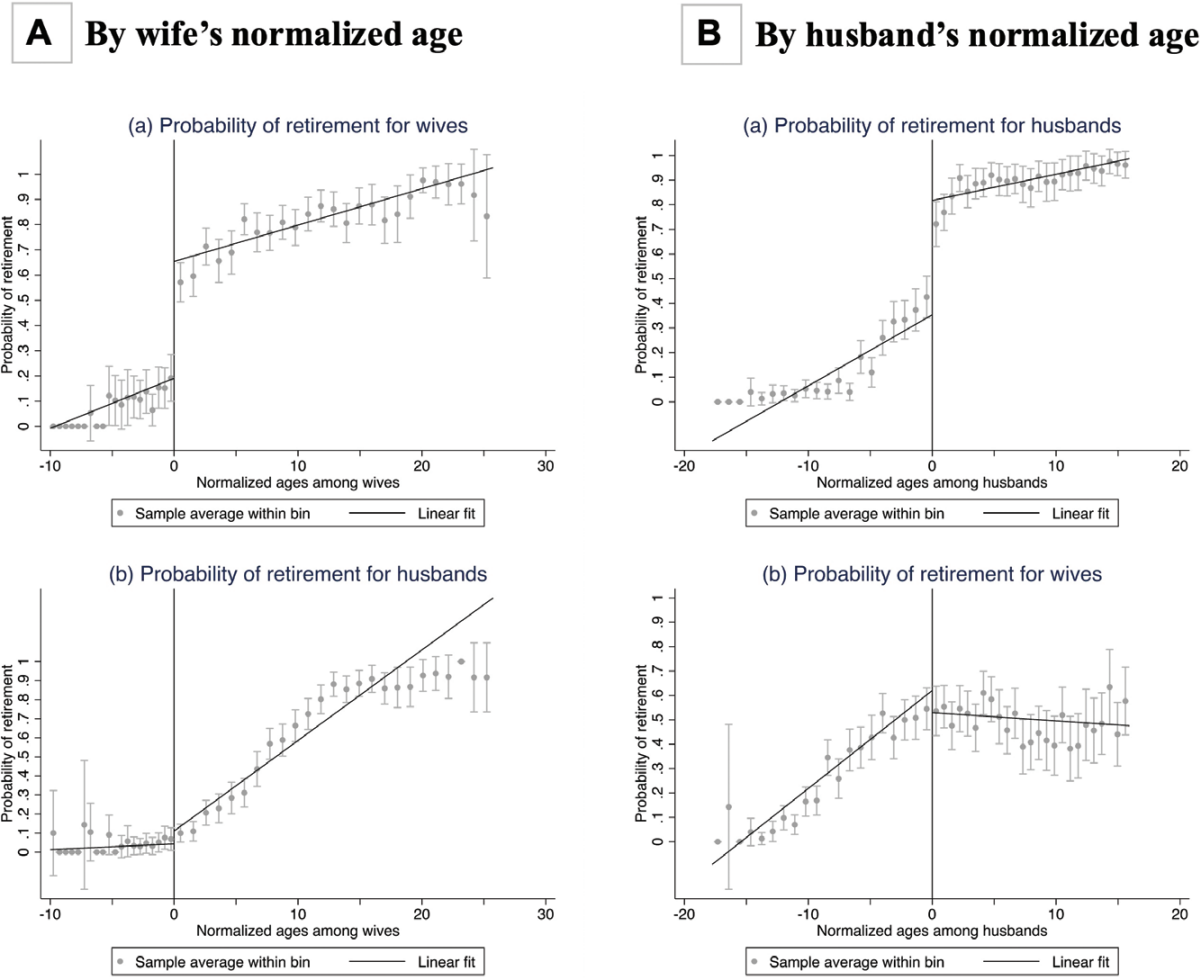
Probability of retirement by wife’s or husband’s normalized age. RDD plots of the probability of (a) self-retirement and (b) spousal retirement for (A) wives and (B) husbands by normalized ages (real age minus 50 for wives; real age minus 60 for husbands), with 95% confidence intervals and a linear fit. We used a uniform kernel, applied individual sampling weights, and set the number of bins as 20 to the left and 25 to the right. RDD = regression discontinuity design.

Fuzzy RDD estimates the causal effect of spousal retirement on disability under the following assumptions: First, for valid causal inferences, retirement probabilities must exhibit discontinuities at the thresholds. Figure 1A shows a significant jump in the probability of a wife’s retirement at the female SRA threshold but no such jump for husbands when their wives reach that threshold. This suggests that age 50 (i.e., the female retirement age) serves as a suitable threshold for discontinuity in wives’ retirement status, and this discontinuity is not contaminated by changes in husbands’ retirement status (i.e., any changes in husbands’ health outcomes at the threshold are solely due to changes in their wives’ retirement status). Similarly, Figure 1B shows a significant jump in the probability of a husband’s retirement at the male SRA threshold but no such jump for wives when their husbands reach that threshold. This likewise suggests that age 60 (i.e., the male retirement age) is a good discontinuity threshold for husbands’ retirement status, and this discontinuity is not contaminated by changes in wives’ retirement status. Our RDD estimations would also be biased if participants had reported false ages to manipulate their eligibility status for retirement. Using a visual assessment of a histogram of age around the retirement threshold (Supplementary Figure 2), we found no evidence of such manipulation.

Second, RDD validity requires that covariates are not correlated with the threshold or outcomes. We included various covariates (participant’s age, the spouse’s age, the quadratic and cubed forms of both participants’ and spouses’ ages, the education levels of participants and their spouses, along with an indicator for ages over 60) in our estimation to enhance efficiency gains (Calonico et al., 2019b). Therefore, we plotted participants’ characteristics against the assignment variable in Supplementary Figure 3 and did not observe discontinuity in these characteristics around the threshold. Finally, we formally tested whether there were systematic differences in individual characteristics (i.e., age, middle school completion, and age over 60 years) that were correlated with the threshold by using observed covariates as outcomes and spousal age as an assignment variable. The results are shown in Supplementary Table 2 and indicate that there is no evidence of systematic differences. Given the non-manipulability of the assignment variable and continuous pretreatment covariates around the threshold, the exogenous assignment rule engenders local randomization in treatment status around the threshold (Imbens & Lemieux, 2008; Moscoe et al., 2015). However, it’s important to note that because the RDD design necessitates a narrow window around retirement for proper identification of causal effects, the effects observed are inherently short term.

Furthermore, we performed several heterogeneous analyses and robustness tests to bolster the reliability of our main results. These analyses were guided by a comprehensive review of pertinent literature (De Winter et al., 2012; Hung et al., 2012; Mehta & Myrskylä, 2017; Ortiz et al., 2022; Scuteri et al., 2011), and were tailored to elucidate the underlying mechanisms central to our outcome of interest. Specifically, we focused on two primary dimensions: the husband’s health status and health behaviors. Within the health status dimension, we further delved into prevalent chronic conditions and psychological well-being. This approach was consistent with our overarching goal of understanding the nuanced effects of retirement on spousal caregiving dynamics.

Therefore, we explored the heterogeneous effect of a wife’s retirement on her husband’s disability by the husband’s retirement status, his diagnosed chronic disease status (diabetes, hypertension, and heart disease), his lifestyle behaviors (smoking, alcohol consumption, physical activity, and social participation), his depressive symptoms and the wife’s educational attainment. Additionally, we performed the following two robustness tests: First, we used different bandwidths of 50%, 75%, 125%, and 150% of the optimal bandwidth. Second, we performed a so-called “donut-hole” estimation to investigate if our results are sensitive to observations close to the threshold by excluding observations around the threshold (Barreca et al., 2016). Moreover, we explored three mechanisms potentially underlying this relationship. These mechanisms fall into the categories of health behaviors (H1), emotions (H2), and diagnosis of diseases (H3). To test H1–H3, we examined the effect of a wife’s retirement on her husband’s health behaviors, emotions, and disease diagnosis. The results are reported in Supplementary Table 5.

## Results

### Summary Statistics

Table 1 presents the summary statistics of the full sample and of the sample stratified by gender. Hypertension was defined as a self-report of hypertension diagnosis by a physician, usage of antihypertensive medication, and/or systolic blood pressure greater than or equal to 140 mm Hg, and/or diastolic blood pressure greater than or equal to 90 mm Hg. Diabetes was defined as a self-report of diabetes diagnosis by physician, being on treatment for diabetes, and/or having a fasting glucose (FPG) level greater than or equal to 126 mg/dL and/or HbA1c > 6.5%. The presence of heart disease was determined via self-reported history obtained through an in-person visit with study personnel via a questionnaire. Depressive symptoms were measured based on the Center for Epidemiological Studies—Depression Scale (CES-D-10) using 10 questions in CHARLS, and a cutoff score of ≥10 (range: 0–30) was used to distinguish participants with depression from those relatively free of depression. Social participation is assigned a value of 1 if the respondent participated in any of 11 specified social activities in the past month and 0 otherwise. Physical activity (PA) was constructed based on the International Physical Activity Questionnaire, and participants with PA score above the highest tertile was defined as physically active (a dichotomous PA variable as 1), and PA score below the highest tertile was defined as physically inactive (a dichotomous PA variable as 0). Current smoking status was categorized as current smoking or noncurrent smoking using the question “Do you smoke cigarettes now?” (yes or no). Excessive alcohol use was defined as more than 14 drinks per week for men and more than 7 drinks per week for women following the National Institute on Alcohol Abuse and Alcoholism guidelines. Alcohol consumption was measured by multiplying the number of days per week that alcohol was consumed by the number of drinks per day, which resulted in the number of drinks per week. Participants who were not in this alcohol consumption range were classified as nonexcessive alcohol use.

**Table 1.** Descriptive Statistics: Full Sample and the Sample Stratified by Gender

Variable	Full sample				Men				Women			
	Mean (*SD*)	Min	Max	*n*	Mean (*SD*)	Min	Max	*n*	Mean (*SD*)	Min	Max	*n*
Outcomes												
ADL score	6.47 (1.54)	6	24	7,227	6.58 (1.94)	6	24	2,983	6.41 (1.30)	6	24	4,244
IADL score	5.48 (1.76)	5	20	7,227	5.41 (1.79)	5	20	2,983	5.52 (1.74)	5	20	4,244
Covariates												
Age	57.17 (7.87)	40	75	7,227	57.93 (7.84)	41	75	2,983	56.64 (7.85)	40	75	4,244
Spouse’s age	57.67 (8.05)	40	75	7,227	55.75 (7.46)	40	75	2,983	59.01 (8.17)	42	75	4,244
Middle school graduate or higher	0.67 (0.47)	0	1	7,227	0.79 (0.41)	0	1	2,983	0.58 (0.49)	0	1	4,244
Spouse middle school graduate or higher	0.73 (0.44)	0	1	7,227	0.72 (0.45)	0	1	2,983	0.74 (0.44)	0	1	4,244
Mechanism												
Chronic disease status												
Hypertension	0.40 (0.49)	0	1	5,620	0.41 (0.49)	0	1	2,326	0.39 (0.49)	0	1	3,294
Diabetes	0.16 (0.37)	0	1	5,311	0.17 (0.38)	0	1	2,208	0.15 (0.36)	0	1	3,103
Heart Disease	0.15 (0.37)	0	1	5,266	0.15 (0.35)	0	1	2,266	0.17 (0.38)	0	1	3,222
Lifestyle behaviors												
Current smoker	0.20 (0.40)	0	1	5,460	0.46 (0.50)	0	1	1,795	0.03 (0.18)	0	1	3,655
Excessive drinker	0.34 (0.47)	0	1	7,221	0.45 (0.50)	0	1	2,979	0.26 (0.44)	0	1	4,242
Physical activity	0.31 (0.46)	0	1	6,303	0.31 (0.46)	0	1	2,575	0.30 (0.46)	0	1	3,728
Mental health status												
Depressive symptoms	0.41 (0.49)	0	1	6,601	0.33 (0.47)	0	1	2,642	0.46 (0.50)	0	1	3,959
Number of unique individuals				3,694				1,601				2,093

*Notes:* ADL = activities of daily living; CHARLS = China Health and Retirement Longitudinal Study; IADL = instrumental activities of living; *SD* = standard deviation. The sample is from the 2011, 2013, 2015, and 2018 waves of CHARLS. This table reports the descriptive statistics for the full sample and the sample stratified by participants’ gender. ADL/IADL scores, age, and spouse age are continuous variables, and middle school graduate or higher and spouse middle school graduate or higher are binary variables.

The average ADL score for men was higher than that for women, both ranging from 6 to 24, whereas women’s average IADL score was higher than that for men (5.52 vs 5.41). The percentage of men who have graduated from middle school surpasses that of women (79.25% vs 57.56%), and the prevalence of diagnosed hypertension (41% vs 39%) and diabetes (17% VS 15%) was also notably elevated among men in contrast to women. Moreover, a significantly higher proportion of men engage in current smoking (46% vs 3%) and excessive alcohol consumption (45% vs 26%) compared to their women counterparts. Conversely, a higher prevalence of depressive symptoms is evident among women when compared to men (46% vs 33%).

### Causal Effect Estimates

Table 2 presents the short-term effects of spousal retirement on individuals’ physical health (see Supplementary Figure 4 for RDD plots). The “conventional” estimates are conventional RDD estimates with a conventional variance estimator (O’Keeffe & Baio, 2016). The “robust” estimates are bias-corrected RDD estimates with a robust variance estimator (Calonico et al., 2014a). We find no evidence that a husband’s retirement status affects his wife’s disability severity. However, we find a significant beneficial effect of wives’ retirement on the severity of ADL among their husbands. The most robust finding is the significant advantageous effect of wives’ retirement on husbands’ ADL score, with a magnitude of 0.644 points (−9.78% relative to baseline). Considering that the mean ADL score among men was 6.58 points, as reported in Table 1, the observed effect size is considerable (around 10% improvement in ADL scores). Table 3 demonstrates the short-term effects of spousal retirement on individuals’ ADL/IADL items. We found a significantly beneficial effect of wives’ retirement on the prevalence of husbands’ difficulty in dressing, bathing, and eating, with substantial magnitudes of 0.075, 0.201, and 0.051 points, respectively. Specifically, a wife’s retirement decreases the probability that her husband experiences difficulty in bathing by 0.201 (robust estimation), suggesting that approximately 20 of every 100 men no longer report any difficulty with bathing following their wives’ retirement.

**Table 2.** Impact of Spousal Retirement on ADL and IADL Disability Severity

Variable	ADL score	IADL score
Wife’s retirement		
Conventional	−0.936^***^	−1.309^***^
	(0.193)	(0.440)
Robust	−0.644^**^	−0.112
	(0.250)	(0.539)
Bandwidth	2.887	2.782
Number of observation	1,387	2,970
Number of unique individuals	1,312	2,084
Husband’s retirement		
Conventional	−0.458	0.897
	(0.686)	(0.974)
Robust	0.780	0.899
	(0.882)	(1.151)
Bandwidth	2.786	4.066
Number of observation	2,814	4,220
Number of unique individuals	733	1,596

*Notes.* ADL = activities of daily living; CHARLS = China Health and Retirement Longitudinal Survey; IADL = instrumental activities of living. The sample is from the 2011, 2013, 2015, and 2018 waves of CHARLS. Covariate-adjusted local linear fuzzy RDD estimation with (robust) and without (conventional) bias-adjustment of point estimation and inference. All standard errors (in parentheses) are clustered with plug-in residuals at the individual level.

^*^ *p < *.1.

^**^ *p* < .05.

^***^ *p* < .01.

**Table 3.** Impact of Spousal Retirement on ADL and IADL Individual Item

Disability Item list	Dressing	Bathing	Eating	Walking across the room	Toileting	Urination	Doing household chores	Preparing hot meals	Shopping	Managing assets	Taking medications
Conventional	−0.085^***^	−0.226^***^	−0.051^***^	−0.044^***^	−0.124	−0.089	−0.039	−0.103^**^	−0.055^**^	−0.161^**^	0.022
	(0.024)	(0.082)	(0.013)	(0.012)	(0.107)	(0.062)	(0.033)	(0.042)	(0.025)	(0.065)	(0.019)
Robust	−0.075^**^	−0.201^**^	−0.051^***^	−0.020	−0.089	−0.099	0.016	−0.036	−0.012	−0.096	0.036
	(0.035)	(0.088)	(0.015)	(0.015)	(0.143)	(0.078)	(0.046)	(0.056)	(0.037)	(0.084)	(0.026)
Bandwidth	2.533	2.698	2.349	2.817	2.465	2.883	2.586	2.655	2.568	2.425	2.158
*N*	2,215	2,215	2,215	2,215	2,182	2,215	4,799	4,790	4,797	4,794	4,797

*Notes*: ADL = activities of daily living; CHARLS = China Health and Retirement Longitudinal Survey; IADL = instrumental activities of living; RDD = regression discontinuity design. The sample is from the 2011, 2013, 2015, and 2018 waves of CHARLS. Covariate-adjusted local linear fuzzy RDD estimation with (robust) and without (conventional) bias-adjustment of point estimation and inference. All standard errors (in parentheses) are clustered with plug-in residuals at the individual level.

^*^ *p* < .1.

^**^ *p* < .05.

^***^ *p* < .01.

### Heterogeneity Analysis

Tables 4 and 5a–c present the heterogeneous effects of a wife’s retirement on her husband’s disability severity, stratified by several key factors. These include the husband’s retirement status, the wife’s level of educational attainment, the husband’s health status encompassing diagnosed chronic diseases and mental well-being, as well as his lifestyle behaviors.

**Table 4.** Impact of a Wife’s Retirement on Her Husband’s Physical Health by the Husband’s Retirement Status and the Wife’s Educational Attainment

Variable	ADL score	IADL score
Husband’s retirement status		
Retired husband		
Conventional	2.724^***^	2.589
	(0.646)	(1.625)
Robust	2.527	2.125
	(1.638)	(1.658)
Bandwidth	1.682	2.361
*N*	709	1,261
Unretired husband		
Conventional	−0.380^***^	−0.348^***^
	−0.146	−0.133
Robust	−0.533^**^	−0.595^***^
	−0.208	−0.189
Bandwidth	2.046	1.803
*N*	678	1709
Wife’s educational attainment		
Wife has not completed middle school		
Conventional	−0.308	−0.769
	(0.345)	(1.332)
Robust	−0.274	1.691
	(0.754)	(1.964)
Bandwidth	2.230	2.698
*N*	459	838
Wife has completed middle school		
Conventional	−1.220^***^	−1.072^**^
	(0.294)	(0.479)
Robust	−1.048^***^	−1.516^***^
	(0.349)	(0.573)
Bandwidth	2.436	2.153
*N*	928	2,132

*Notes:* ADL = activities of daily living; CHARLS = China Health and Retirement Longitudinal Survey; IADL = instrumental activities of living; RDD = regression discontinuity design. The sample is from the 2011, 2013, 2015, and 2018 waves of CHARLS. Covariate-adjusted local linear fuzzy RDD estimation with (robust) and without (conventional) bias-adjustment of point estimation and inference. All standard errors (in parentheses) are clustered with plug-in residuals at the individual level. The point estimates are calculated based on bandwidths selected using a data-driven method. Effective bandwidths are presented in the table.

^*^ *p* < .1.

^**^ *p* < .05.

^***^ *p* < .01.

**Table 5.** Impact of a Wife’s Retirement on Her Husband’s Physical Health by the Husband’s Health Conditions (Diagnosed Chronic Disease Status Including Diabetes, Hypertension and Heart Disease, and Mental Health Status Including Depressive Symptoms)

Variable	Husband’s diabetes status		Husband’s hypertension status		Husband’s heart disease status		Husband’s depressive symptoms	
	ADL score	IADL score	ADL score	IADL score	ADL score	IADL score	ADL score	IADL score
Yes								
Conventional	−1.536	−0.160	−1.271^***^	−1.201^**^	−1.442^**^	−2.385	−3.124^***^	−0.722^***^
	(1.013)	(0.325)	(0.443)	(0.549)	(0.616)	(1.631)	−1.143	−0.248
Robust	−1.658	−0.777^*^	−1.628^***^	−1.252^**^	−1.530^**^	−3.103	−2.622^*^	−0.649^*^
	(1.356)	(0.411)	(0.595)	(0.607)	(0.761)	(2.014)	−1.387	−0.366
Bandwidth	3.082	2.287	3.397	3.616	3.682	3.520	2.754	2.665
*N*	218	353	517	860	327	449	499	626
No								
Conventional	−0.561^***^	−1.099^***^	−0.146	−1.027^**^	−0.876^**^	−0.504^***^	−1.457^***^	−0.354^*^
	(0.138)	(0.359)	(0.133)	(0.417)	(0.390)	(0.185)	−0.559	−0.213
Robust	−0.539^***^	−0.816^*^	−0.274^*^	−0.586	−0.615	−0.282	−0.734	−0.445^*^
	(0.171)	(0.423)	(0.156)	(0.527)	(0.491)	(0.232)	−0.797	−0.242
Bandwidth	2.530	2.994	1.949	2.485	2.463	3.092	2.725	2.15
*N*	895	1,802	594	1,293	756	1,553	1,572	3,100

*Notes:* ADL = activities of daily living; CHARLS = China Health and Retirement Longitudinal Survey; IADL = instrumental activities of living; RDD = regression discontinuity design. The sample is from the 2011, 2013, 2015, and 2018 waves of CHARLS. Covariate-adjusted local linear fuzzy RDD estimation with (robust) and without (conventional) bias-adjustment of point estimation and inference. All standard errors (in parentheses) are clustered with plug-in residuals at the individual level. The point estimates are calculated based on bandwidths selected using a data-driven method. Effective bandwidths are presented in the table.

^*^ *p* < .1.

^**^ *p* < .05.

^***^ *p* < .01.

Table 4 shows that the health of unretired husbands improved following their wives’ retirement, with these men experiencing sizable reductions of 0.533 points in average ADL score and 0.595 points in average IADL score. Furthermore, larger effects were observed for the husbands or wives who had completed middle school, with these men experiencing reductions of 1.048 points in average ADL score and 1.516 points in average IADL score. However, the effect of wives’ retirement on retired husbands was not robust. The results in Tables 5 and 6 present that the positive spillover effect of a wife’s retirement is robust among several subgroups. The impact of a wife’s retirement on her husband’s disability was notably stronger among husbands with diagnosed hypertension, heart disease, and depressive symptoms, displaying substantial reductions of 1.628 (−23.829% relative to baseline), 1.530 (−23.394% relative to baseline), and 2.622 points (−40.444% relative to baseline) in average ADL score, respectively. Similarly, husbands who engaged in light PA abstained from social participation and refrained from excessive drinking experienced sizable decreases of 1.120 (−20.584% relative to baseline), 1.470 (−24.979% relative to baseline), and 1.523 points (−27.328% relative to baseline) in average IADL score, respectively.

**Table 6.** Impact of a Wife’s Retirement on Her Husband’s Physical Health by the Husband’s Lifestyle Behaviors (Smoking, Alcohol Consumption, Physical Activity and Social Participation)

Variable	Husband’s current smoking behavior		Husband’s current excessive drinking behavior		Husband’s physical activity behavior		Husband’s social activity participation	
	ADL score	IADL score	ADL score	IADL score	ADL score	IADL score	ADL score	IADL score
Yes								
Conventional	0.044	−1.945	−0.608^***^	−0.210^**^	2.061^***^	−1.175	−0.924^**^	−1.062
	(0.289)	(1.697)	(0.225)	(0.088)	(0.691)	(1.137)	(0.413)	(0.696)
Robust	0.103	−2.195	−0.537^*^	−0.178	1.876^*^	−0.294	−0.893^*^	−1.069
	(0.363)	(2.152)	(0.315)	(0.125)	(1.046)	(1.372)	(0.539)	(0.832)
Bandwidth	2.854	3.421	2.891	2.484	1.816	2.380	3.495	2.806
*N*	427	889	583	1,135	332	664	638	1,336
No								
Conventional	−0.988^***^	−0.889^***^	−0.710^***^	−1.749^***^	−0.973^**^	−1.116^***^	−0.884^***^	−1.298^***^
	(0.323)	(0.281)	(0.249)	(0.559)	(0.403)	(0.329)	(0.198)	(0.340)
Robust	−0.785^*^	−0.812^**^	−0.806^***^	−1.523^**^	−0.759	−1.120^***^	−0.553^***^	−1.470^***^
	(0.455)	(0.353)	(0.287)	(0.652)	(0.520)	(0.391)	(0.209)	(0.388)
Bandwidth	3.657	3.146	2.124	2.650	3.533	2.726	2.675	2.022
*N*	573	1,068	804	1,558	781	1,491	475	819

*Notes*: ADL = activities of daily living; CHARLS = China Health and Retirement Longitudinal Survey; IADL = instrumental activities of living; RDD = regression discontinuity design. The sample is from the 2011, 2013, 2015, and 2018 waves of CHARLS. Covariate-adjusted local linear fuzzy RDD estimation with (robust) and without (conventional) bias-adjustment of point estimation and inference. All standard errors (in parentheses) are clustered with plug-in residuals at the individual level. The point estimates are calculated based on bandwidths selected using a data-driven method. Effective bandwidths are presented in the table.

^*^ *p < *.1.

^**^ *p* < .05.

^***^ *p* < .01.

### Robustness Checks

The results of using different bandwidths (shown in Supplementary Table 3) are similar to our main findings. For 50% of the optimal bandwidth, the effects of wife’s retirement on her husband’s IADL disability severity are not significant at the traditional levels, which might be because of a 50% drop in the effective observations, though the coefficient is comparable to that observed with the optimal bandwidth. For the “donut-hole” estimation, we specifically performed three sensitivity analyses in which we excluded 1 month, 2 months, and 3 months of observations on either side of the threshold. The results of these analyses (Supplementary Table 4) are also largely consistent with our main results.

### Mechanism Exploration

We found that a wife’s retirement did not improve her husband’s health behaviors (Supplementary Table 5): It had no significant effect on behaviors of current smoking, excessive drinking, whether is physically active, or his level of social participation. Second, we found robust evidence that the number of depressed days experienced by husbands fell—and their number of happy days increased—after their wives’ retirement. These findings indicate that a wife’s retirement has a positive effect on her husband’s emotional well-being. Though we noted that disability may contribute to alterations in mental health status (i.e., reverse causality; Chen et al., 2012), further research is warranted to examine the causal relationship between emotional status and the severity of disability.

## Discussion

Using data from the 2011, 2013, 2015, and 2018 waves of CHARLS, this study examined the spillover effect of retirement on spouses’ disability severity among Chinese urban residents for the first time. By utilizing legal retirement ages as a source of exogenous variation in retirement and applying a fuzzy RDD, we found gender-asymmetric effects of retirement: Wives’ retirement reduced ADL and IADL disability in their husbands, whereas husbands’ retirement had no such effect for their wives. The beneficial spillover effect from a wife’s retirement likely results from reducing her husband’s negative emotions and increasing his positive emotions. Additionally, we found heterogeneous effects of a wife’s retirement on her husband’s health by the husband’s retirement status, his diagnosed chronic disease status (diabetes, hypertension, and heart disease), his lifestyle behaviors (smoking, alcohol consumption, PA, and social participation), his depressive symptoms, and the wife’s educational attainment. The favorable spillover effect is most robust when the husband is unretired or when the wife has higher educational attainment. Given that educational attainment significantly influences income sustainability (Carlson & McChesney, 2015) and marital satisfaction (Zhang & Liang, 2023), our findings imply that both financial stability and the quality of the marital relationship may be pivotal factors in the association between a wife’s retirement and her husband’s health outcomes.

The spillover effect of retirement on health might be context dependent and driven by differences in gender roles and family structures. Only two studies have specifically investigated the spillover effect of retirement on disability in China and the United States (Newmyer et al., 2023; Zang, 2020). Our findings align with Zang (2020), which utilized the same data source and disability definition, and revealed that a husband’s retirement did not affect his wife’s functional limitation. However, Zang (2020) solely concentrated on the influence of husbands’ retirement on their wives’ health, neglecting the spillover effect of wives’ retirement on their husbands’ disability status. The health effects of spousal retirement exhibit very different patterns for men and women, indicating that ignoring the gender difference in spillover effects would provide misleading evidence. Moreover, the severity and individual items of functional limitations were not explored in Zang’s (2020) research. Conversely, another study using U.S. data observed a negative effect on husbands’ disability following their spouses’ retirement, with a significant increase in both the ADL (0.85, *p* < .01) and IADL (0.67, *p* < .01) measures of functional limitations (Newmyer et al., 2023). This increase was also observed among unretired husbands. This discrepancy may be attributed to stricter gender roles prevailing in China as compared to the United States. In China, women typically shoulder family caregiving responsibilities and a heavier housework workload, whereas in the United States, men may also be likely to experience negative health outcomes due to spousal retirement as economic hardship emerges (Neppl et al., 2016). In contexts such as Europe, gender roles can be more flexible than in the United States, which makes spousal spillover effects of retirement on health related even less to gender (T. Müller & M. Shaikh, 2018). Additionally, consistent with our study, no notable spillover effects on disability were detected for women in all previous research (Newmyer et al., 2023; Zang, 2020), which demonstrated that retirement’s spillover effects on disability are more prominent among women than men regardless of the contexts (Liu, 2021).

The findings of this study contribute empirical evidence concerning the gender-asymmetric spillover short-term effects of spousal retirement on disability and further our understanding of the factors that influence ADL/IADL disability. Based on the theory of “Retired Husband Syndrome” (Johnson, 1984), women’s health might suffer when their husband retires as they unequally bear the increased burden of the caretaking of their home, their spouse, and themselves; By contrast, the family stress model proposes that men may be the most likely to experience negative health outcomes due to spousal retirement as economic hardship might manifest as more damaging for men in contrast to women (Masarik et al., 2016). Therefore, the effects of spousal retirement on ADL/IADL disability severity may also vary by gender due to gender-based differences in social norms and expectations (Feld et al., 2010). There are important underlying gender differences in mortality and morbidity, which can be explained by both biological and behavioral factors (Schünemann et al., 2017). ADL disability is an especially important issue for women, who are expected to spend a larger proportion of life in poor health than men (Luy & Minagawa, 2014). Because the retirement age differs for men and women in many countries (e.g., in China men retire at age 60 whereas women retire at age 50 or 55), the effects of retirement on ADL/IADL disability may also be modified by gender (Kozakai et al., 2000). Furthermore, in accordance with historical gender roles in many settings, men are more likely to consider work as central to their lives and tend to be more continuously employed up to retirement, whereas women generally focus relatively more on the home. Men may, therefore, be more likely to perceive job loss as a social failure and find retirement more stressful than women (Van Solinge, 2007). At the same time, married men benefit more from spousal care than married women in a U.S. cohort (Noël-Miller, 2010), reflecting broader findings that wives are more frequently identified as sole caregivers under the theoretical frameworks of gender role socialization and the gendered division of domestic labor. Because retirement is a key determinant of various objective and subjective health outcomes (Dave et al., 2008), there is a growing consensus that retirement reforms should be carefully designed to account for their potential social and economic effects (Frimmel & Pruckner, 2020). The insights into gender heterogeneity in the effects of spousal retirement yielded by this study may help policy-makers design differentiated policies for men and women.

Moreover, we observed a more pronounced effect of wives’ retirement on the health of husbands who had not retired. Although gendered caregiving roles could persist following a spouse’s retirement, husbands who have not retired may be less affected by the economic strains brought on by their spouse’s retirement. This explains why we exclusively observed a positive impact of wives’ retirement on the health of husbands who had not retired. Our heterogeneity analysis further substantiated this mechanism, indicating that a wife’s retirement might enhance her husband’s health by potentially increasing the likelihood of medical diagnosis for chronic conditions. Husbands with heart disease, diabetes, or hypertension might exhibit more frequent visits to doctors, thus preemptively averting the occurrence of chronic diseases. Additionally, this approach would foster early detection, slow disease progression, reduce complications, and ultimately lead to an improved quality of life and diminished prevalence or severity of disability (Bauer et al., 2014; Grimby et al., 1988; Verbrugge & Jette, 1994; W. W. Hung et al., 2012).

Notably, more significant effects were evident in husbands whose wives had completed at least a middle school education. These men experienced reductions of 1.048 points in the average ADL score and 1.516 points in the average IADL score. The finding of more substantial positive spillover effects from the retirement of wives with middle school education or higher is reasonable because more educated women might face fewer financial constraints and could experience increased marital satisfaction after retirement compared with less educated women (Heaton, 2002; Kridahl & Duvander, 2023; Mirecki et al., 2013), particularly in a cultural context like China where traditional gender roles were held (Yu & Liu, 2021).

In further analysis, we observed that the effects of wives’ retirement on husbands’ disability might be observed through reducing negative emotions and increasing the days of happiness. Our findings are consistent with previous literature, showing that a wife’s retirement may improve her husband’s mental health (Atalay & Zhu, 2018; Chen et al., 2020, 2021; Zang, 2020). More specifically, men with retired wives are more likely to engage in voluntary work, participate in frequent socialization, retire, and be active members of clubs or associations among the Australian population, all of which may help improve mental health among older husbands (Atalay & Zhu, 2018). We presented compelling evidence indicating that within the cohort of husbands diagnosed with hypertension, diabetes, depressive symptoms, and engaged in light PA or abstained from social participation, the beneficial impact of wives’ retirement on their well-being would be notably amplified. This effect may stem from husbands gaining increased access to spousal care, encompassing crucial support in accessing medical consultations or examinations. This phenomenon holds particular significance within the Chinese societal framework, where women typically assume primary caregiving roles for their spouses (Park et al., 2020). However, different from Chen’s research in China (Chen, 2022), we did not observe a direct causal relationship between a wife’s retirement and her husband’s health behaviors. The lack of observed effect could be the result of the disparate measurements of the same health behavior, relatively short-term investigation, and small sample in the analysis.

Although this study has important findings, future research is needed to address some limitations. First, the RDD is limited in terms of external validity. The findings of this study apply specifically to urban wage earners who are close in age to a relevant policy threshold, such as the SRA in China. After dropping observations with missing variables, *t*-tests and χ^2^ tests show that the included sample had a lower average age and higher educational attainment (Supplementary Table 1). This discrepancy may stem from factors such as urban residents from rural areas or with lower educational attainment encountering difficulty securing employment, thus leading to missing data pertaining to work status and retirement. Additionally, it is noteworthy that urban residents originating from rural areas tend to possess lower educational backgrounds. Given the constraints imposed on the age range of the studied sample, the generalizability of our conclusions to individuals who opt for voluntary retirement at substantially younger ages may be limited. Moreover, most Western countries offer re-employment policies under which individuals can still work after reaching retirement age (Zhong & Ma, 2016). China’s institutional settings lead to a much higher number of “compliers,” which refers to people who retire at the legal retirement age and do not work thereafter (Fang & Shi, 2022). These contextual factors could have implications for external validity. Therefore, additional studies should be undertaken to investigate the effects of specific interventions related to spousal retirement in a variety of country contexts. Second, the identification strategy of RDD in this study only allows estimation in the short term, and it is not fully experimental. Previous study has provided evidence that retirement has positive effects on objective health measures, but these effects take time to occur using U.S. data (Gorry et al., 2018). Moreover, another research also observed that retirement had no short-term effects on mental health, but had a large negative longer-term impact in 10 European countries (Heller-Sahlgren, 2017). By contrast, some researchers found that the positive psychological effects of retirement were temporal and became insignificant in longer post-retirement periods among Japanese men (Okamoto & Kobayashi, 2022). Therefore, future research is needed to examine the long-term health causal effects of spousal retirement. Finally, it is important to acknowledge that the outcomes obtained from multiple tests may not be flawless. However, we aim for our paper to serve as an initial foundation, catalyzing future investigations that can furnish more comprehensive and resilient evidence regarding the intergenerational health implications of retirement.

Our findings carry significant implications for various stakeholders and should inform policy evaluations regarding adjustments to the retirement age. Although our results do not explicitly endorse an earlier retirement age for women, they highlight potential economic and health benefits associated with women’s retirement, including potential reductions in healthcare costs for their spouses. This consideration could influence debates on whether to raise or equalize the retirement age for women, contributing to broader discussions on domestic responsibilities within marriages. Future research should focus on strategies that support gender equity in balancing work and family life, ensuring women are not inadvertently disadvantaged in their careers or workforce contributions. Moreover, our heterogeneity analysis underscores the urgent need to educate both men and women on maintaining healthy lifestyles and mental wellness to mitigate health issues during retirement. With China facing a rapidly aging population, addressing the physical independence and emotional well-being of older adults is crucial to promoting healthy aging.

## Conclusion

Our findings contribute empirical evidence concerning the gender-asymmetric spillover short-term effects of spousal retirement on disability and further our understanding of the factors that influence ADL/IADL disability. Specifically, we find that wives’ retirement reduced ADL and IADL disability in their husbands, whereas husbands’ retirement had no such effect on their wives. Moreover, we propose and provide supporting evidence that a wife’s retirement might have beneficial effects on her husband’s disability severity, possibly through reducing his negative emotions and improving mental health. We further highlight the heterogeneous impacts retirement can have on spousal health, which underscores the need for tailored interventions and policies that account for the distinct experiences of men and women in relation to retirement and disability. Additionally, as we show here, older adults’ health is linked to spousal spillover effects, which highlights an important need for the consideration of the family unit rather than just the retiree in shaping policy decisions related to older and retiring adults.

## Supplementary Material

igae074_suppl_Supplementary_Material

## Data Availability

This study has not been preregistered. Publicly available data sets were analyzed in this study. These data can be found at http://charls.pku.edu.cn/en and https://share-eric.eu/data/.

## References

[CIT0001] Adams, G. A., Prescher, J., Beehr, T. A., & Lepisto, L. (2002). Applying work-role attachment theory to retirement decision-making. International Journal of Aging & Human Development, 54(2), 125–137. 10.2190/JRUQ-XQ2N-UP0A-M432 12054271

[CIT0002] Atalay, K., & Zhu, R. (2018). The effect of a wife’s retirement on her husband’s mental health. Applied Economics, 50(43), 4606–4616. 10.1080/00036846.2018.1458198

[CIT0003] Barreca, A. I., Lindo, J. M., & Waddell, G. R. (2016). Heaping‐induced bias in regression‐discontinuity designs. Economic Inquiry, 54(1), 268–293. 10.1111/ecin.12225

[CIT0004] Barry, L. C., Allore, H. G., Bruce, M. L., & Gill, T. M. (2009). Longitudinal association between depressive symptoms and disability burden among older persons. Journals of Gerontology, Series A: Biological Sciences and Medical Sciences, 64(12), 1325–1332. 10.1093/gerona/glp135 19776217 PMC2773818

[CIT0005] Bauer, U. E., Briss, P. A., Goodman, R. A., & Bowman, B. A. (2014). Prevention of chronic disease in the 21st century: Elimination of the leading preventable causes of premature death and disability in the USA. Lancet (London, England), 384(9937), 45–52. 10.1016/S0140-6736(14)60648-6 24996589

[CIT0006] Bertoni, M., & Brunello, G. (2017). Pappa Ante Portas: The effect of the husband’s retirement on the wife’s mental health in Japan. Social Science & Medicine (1982), 175, 135–142. 10.1016/j.socscimed.2017.01.012 28088619

[CIT0007] Calonico, S., Cattaneo, M. D., Farrell, M. H., & Titiunik, R. (2017). rdrobust: Software for regression-discontinuity designs. Stata Journal, 17(2), 372–404. 10.1177/1536867x1701700208

[CIT0008] Calonico, S., Cattaneo, M. D., Farrell, M. H., & Titiunik, R. (2019a). Regression discontinuity designs using covariates. Review of Economics and Statistics, 101(3), 442–451. 10.1162/rest_a_00760

[CIT0009] Calonico, S., Cattaneo, M. D., Farrell, M. H., & Titiunik, R. (2019b). Regression discontinuity designs using covariates. Review of Economics and Statistics, 101(3), 442–451. 10.1162/rest_a_00760

[CIT0010] Calonico, S., Cattaneo, M. D., & Titiunik, R. (2014a). Robust data-driven inference in the regression-discontinuity design. Stata Journal, 14(4), 909–946. 10.1177/1536867x1401400413

[CIT0011] Calonico, S., Cattaneo, M. D., & Titiunik, R. (2014b). Robust nonparametric confidence intervals for regression‐discontinuity designs. Econometrica, 82(6), 2295–2326. 10.3982/ecta11757

[CIT0012] Carlson, R., & McChesney, C. (2015). Income sustainability through educational attainment. Exchange, 4(1), 44. 10.11114/jets.v3i1.508

[CIT0013] Carnethon, M. R., Kinder, L. S., Fair, J. M., Stafford, R. S., & Fortmann, S. P. (2003). Symptoms of depression as a risk factor for incident diabetes: Findings from the National Health and Nutrition Examination Epidemiologic Follow-up Study, 1971–1992. American Journal of Epidemiology, 158(5), 416–423. 10.1093/aje/kwg172 12936896

[CIT0015] Chen, C.-M., Mullan, J., Su, Y.-Y., Griffiths, D., Kreis, I. A., & Chiu, H.-C. (2012). The longitudinal relationship between depressive symptoms and disability for older adults: A population-based study. Journals of Gerontology, Series A: Biological Sciences and Medical Sciences, 67(10), 1059–1067. 10.1093/gerona/gls074 22454375

[CIT0016] Chen, S., Geldsetzer, P., & Bärnighausen, T. (2020). The causal effect of retirement on stress in older adults in China: A regression discontinuity study. SSM—Population Health, 10, 100462. 10.1016/j.ssmph.2019.100462 32083164 PMC7016446

[CIT0017] Chen, S., Hao, Z., & Bärnighausen, T. (2021). The effects of a wife’s retirement on her husband’s mental health among older adults in China. Applied Economics Letters, 29, 1891–1896. 10.1080/13504851.2021.1965081

[CIT0018] Chen, X. (2022). The impact of spousal and own retirement on health: Evidence from urban China. World Development, 159, 106025. 10.1016/j.worlddev.2022.106025

[CIT0019] Coe, N. B., & Zamarro, G. (2011). Retirement effects on health in Europe. Journal of Health Economics, 30(1), 77–86. 10.1016/j.jhealeco.2010.11.002 21183235 PMC3972912

[CIT0020] d’Orsi, E., Xavier, A. J., Steptoe, A., de Oliveira, C., Ramos, L. R., Orrell, M., Demakakos, P., & Marmot, M. G. (2014). Socioeconomic and lifestyle factors related to instrumental activity of daily living dynamics: Results from the English Longitudinal Study of Ageing. Journal of the American Geriatrics Society, 62(9), 1630–1639. 10.1111/jgs.12990 25243677

[CIT0021] Dave, D., Rashad, I., & Spasojevic, J. (2008). The effects of retirement on physical and mental health outcomes. Southern Economic Journal, 75(2), 497–523. 10.1002/j.2325-8012.2008.tb00916.x

[CIT0022] De Winter, C., Bastiaanse, L., Hilgenkamp, T., Evenhuis, H., & Echteld, M. (2012). Cardiovascular risk factors (diabetes, hypertension, hypercholesterolemia and metabolic syndrome) in older people with intellectual disability: Results of the HA-ID study. Research in Developmental Disabilities, 33(6), 1722–1731. 10.1016/j.ridd.2012.04.010 22699246

[CIT0023] Deng, Y., & Paul, D. R. (2018). The relationships between depressive symptoms, functional health status, physical activity, and the availability of recreational facilities: A rural-urban comparison in middle-aged and older Chinese adults. International Journal of Behavioral Medicine, 25(3), 322–330. 10.1007/s12529-018-9714-3 29498014

[CIT0024] Ebeid, M., & Oguzoglu, U. (2023). Short‐term effect of retirement on health: Evidence from nonparametric fuzzy regression discontinuity design. Health Economics, 32(6), 1323–1343. 10.1002/hec.4669 36862580

[CIT0025] Eibich, P., & Siedler, T. (2020). Retirement, intergenerational time transfers, and fertility. European Economic Review, 124, 103392. 10.1016/j.euroecorev.2020.103392

[CIT0026] Fang, L., & Shi, R. (2022). Comparative analysis of the effects of retirement on health status of older adulthood. International Journal of Environmental Research and Public Health, 19(16), 9957. 10.3390/ijerph19169957 36011591 PMC9407978

[CIT0027] Feld, S., Dunkle, R. E., Schroepfer, T., & Shen, H.-W. (2010). Does gender moderate factors associated with whether spouses are the sole providers of IADL care to their partners? Research on Aging, 32(4), 499–526. 10.1177/0164027510361461 21818168 PMC3148766

[CIT0028] Ferrucci, L., Guralnik, J. M., Pahor, M., Corti, M. C., & Havlik, R. J. (1997). Hospital diagnoses, Medicare charges, and nursing home admissions in the year when older persons become severely disabled. Journal of the American Medical Association, 277(9), 728–734. 10.1001/jama.1997.03540330050034 9042845

[CIT0029] Fletcher, J., & Marksteiner, R. (2017). Causal spousal health spillover effects and implications for program evaluation. American Economic Journal: Economic Policy, 9(4), 144–166. 10.1257/pol.20150573 30057688 PMC6063372

[CIT0030] Frimmel, W., & Pruckner, G. J. (2020). Retirement and healthcare utilization. Journal of Public Economics, 184, 104146. 10.1016/j.jpubeco.2020.104146

[CIT0031] Gao, M., Sa, Z., Li, Y., Zhang, W., Tian, D., Zhang, S., & Gu, L. (2018). Does social participation reduce the risk of functional disability among older adults in China? A survival analysis using the 2005–2011 waves of the CLHLS data. BMC Geriatrics, 18(1), 1–13. 10.1186/s12877-018-0903-3 30241507 PMC6151053

[CIT0032] Gong, J., Wang, G., Wang, Y., Chen, X., Chen, Y., Meng, Q., Yang, P., Yao, Y., & Zhao, Y. (2022). Nowcasting and forecasting the care needs of the older population in China: Analysis of data from the China Health and Retirement Longitudinal Study (CHARLS). Lancet Public Health, 7(12), e1005–e1013. 10.1016/S2468-2667(22)00203-1 36423656 PMC9741660

[CIT0033] Goodman, C. R., & Shippy, R. A. (2002). Is it contagious? Affect similarity among spouses. Aging & Mental Health, 6(3), 266–274. 10.1080/13607860220142431 12217095

[CIT0034] Gorry, A., Gorry, D., & Slavov, S. N. (2018). Does retirement improve health and life satisfaction? Health Economics, 27(12), 2067–2086. 10.1002/hec.3821 30141568

[CIT0035] Gregg, E. W., Beckles, G., Williamson, D. F., Leveille, S. G., Langlois, J. A., Engelgau, M. M., & Narayan, K. (2000). Diabetes and physical disability among older US adults. Diabetes Care, 23(9), 1272–1277. 10.2337/diacare.23.9.1272 10977018

[CIT0036] Grimby, G., Finnstam, J., & Jette, A. (1988). On the application of the WHO handicap classification in rehabilitation. Scandinavian Journal of Rehabilitation Medicine, 20(3), 93–98. 2973124

[CIT0037] Hajek, A., & König, H.-H. (2016). Longitudinal predictors of functional impairment in older adults in Europe—Evidence from the Survey of Health, Ageing and Retirement in Europe. PLoS One, 11(1), e0146967. 10.1371/journal.pone.0146967 26784698 PMC4718586

[CIT0038] Hao, Z., Cheng, H., Bärnighausen, T., & Chen, S. (2024). The effects of parental retirement on adult children’s health: Evidence from China. Health Economics, 33(1), 12–20. 10.1002/hec.4767 37858318

[CIT0039] Heaton, T. B. (2002). Factors contributing to increasing marital stability in the United States. Journal of Family Issues, 23(3), 392–409. 10.1177/0192513x02023003004

[CIT0040] Heller-Sahlgren, G. (2017). Retirement blues. Journal of Health Economics, 54, 66–78. 10.1016/j.jhealeco.2017.03.007 28505541

[CIT0041] Henkens, K., van Solinge, H., & Gallo, W. T. (2008). Effects of retirement voluntariness on changes in smoking, drinking and physical activity among Dutch older workers. European Journal of Public Health, 18(6), 644–649. 10.1093/eurpub/ckn095 18927184 PMC2727140

[CIT0042] Hubert, H. B., Bloch, D. A., & Fries, J. F. (1993). Risk factors for physical disability in an aging cohort: The NHANES I Epidemiologic Followup Study. Journal of Rheumatology, 20(3), 480–488. 8478855

[CIT0043] Hung, W. W., Ross, J. S., Boockvar, K. S., & Siu, A. L. (2012). Association of chronic diseases and impairments with disability in older adults: A decade of change? Medical Care, 50(6), 501–507. 10.1097/MLR.0b013e318245a0e0 22584885 PMC3353149

[CIT0044] Imbens, G. W., & Lemieux, T. (2008). Regression discontinuity designs: A guide to practice. Journal of Econometrics, 142(2), 615–635. 10.1016/j.jeconom.2007.05.001

[CIT0099] Jette, A. M. (2006). Toward a common language for function, disability, and health. *Physical Therapy*, 86(5), 726–734. 10.1093/ptj/86.5.726 16649895

[CIT0045] Jette, A. M. (2021). Global prevalence of disability and need for rehabilitation. Physical Therapy, 101(2), pzab004. 10.1093/ptj/pzab004 33556184

[CIT0046] Johnson, C. C. (1984). The retired husband syndrome. Western Journal of Medicine, 141(4), 542–545. 6506693 PMC1021891

[CIT0047] Johnson, J. V., & Hall, E. M. (1988). Job strain, work place social support, and cardiovascular disease: A cross-sectional study of a random sample of the Swedish working population. American Journal of Public Health, 78(10), 1336–1342. 10.2105/ajph.78.10.1336 3421392 PMC1349434

[CIT0048] Kaneko, Y., Motohashi, Y., Sasaki, H., & Yamaji, M. (2007). Prevalence of depressive symptoms and related risk factors for depressive symptoms among elderly persons living in a rural Japanese community: A cross-sectional study. Community Mental Health Journal, 43(6), 583–590. 10.1007/s10597-007-9096-5 17619147

[CIT0049] Katz, S., Ford, A. B., Moskowitz, R. W., Jackson, B. A., & Jaffe, M. W. (1963). Studies of illness in the aged. The index of ADL: A standardized measure of biological and psychosocial function. Journal of the American Medical Association, 185, 914–919. 10.1001/jama.1963.03060120024016 14044222

[CIT0050] Kozakai, R., Tsuzuku, S., Yabe, K., Ando, F., Niino, N., & Shimokata, H. (2000). Age-related changes in gait velocity and leg extension power in middle-aged and elderly people. Journal of Epidemiology, 10(1 Suppl), S77–81. 10.2188/jea.10.1sup_77 10835832

[CIT0051] Kridahl, L., & Duvander, A. Z. (2023). Relationship satisfaction and money pooling among older working and retired couples in Sweden. Family Relations, 73(2), 1278–1295. 10.1111/fare.12919

[CIT0052] Lawton, M. P., & Brody, E. M. (1969). Assessment of older people: self-maintaining and instrumental activities of daily living. Gerontologist, 9(3), 179–186. 10.1093/geront/9.3_Part_1.179 5349366

[CIT0054] Lee, Y.-H., Lu, P., Chang, Y.-C., Shelley, M., Lee, Y.-T., & Liu, C.-T. (2021). Associations of alcohol consumption status with activities of daily living among older adults in China. Journal of Ethnicity in Substance Abuse, 20(3), 428–443. 10.1080/15332640.2019.1664961 31530097

[CIT0055] Lei, X., & Liu, H. (2018). Gender difference in the impact of retirement on cognitive abilities: Evidence from urban China. Journal of Comparative Economics, 46(4), 1425–1446. 10.1016/j.jce.2018.01.005

[CIT0056] Li, H., Shi, X., & Wu, B. (2016). The retirement consumption puzzle revisited: Evidence from the mandatory retirement policy in China. Journal of Comparative Economics, 44(3), 623–637. 10.1016/j.jce.2015.06.001

[CIT0057] Li, J., Lin, S., Yan, X., Pei, L., & Wang, Z. (2022). Adverse childhood experiences and trajectories of ADL disability among middle-aged and older adults in China: Findings from the CHARLS Cohort Study. Journal of Nutrition, Health & Aging, 26(12), 1034–1041. 10.1007/s12603-022-1863-z 36519765

[CIT0058] Liu, J. (2021). Spillover effects of retirement on physical and mental health of a spouse or partner: Do gender and sexuality matter? Innovation in Aging, 5(Suppl_1), 528–528. 10.1093/geroni/igab046.2036

[CIT0059] Luy, M., & Minagawa, Y. (2014). Gender gaps—Life expectancy and proportion of life in poor health. Health Reports, 25(12), 12–19. 10.1093/eurpub/cku211 25517936

[CIT0060] Man, W., Wang, S., & Yang, H. (2021). Exploring the spatial-temporal distribution and evolution of population aging and social-economic indicators in China. BMC Public Health, 21(1), 966. 10.1186/s12889-021-11032-z 34020620 PMC8140474

[CIT0061] Masarik, A. S., Martin, M. J., Ferrer, E., Lorenz, F. O., Conger, K. J., & Conger, R. D. (2016). Couple resilience to economic pressure over time and across generations. Journal of Marriage and the Family, 78(2), 326–345. 10.1111/jomf.12284 27019520 PMC4806389

[CIT0062] Mehta, N., & Myrskylä, M. (2017). The population health benefits of a healthy lifestyle: Life expectancy increased and onset of disability delayed. Health Affairs, 36(8), 1495–1502. 10.1377/hlthaff.2016.1569 PMC5775051 28724530

[CIT0063] Meyler, D., Stimpson, J. P., & Peek, M. K. (2007). Health concordance within couples: A systematic review. Social Science & Medicine (1982), 64(11), 2297–2310. 10.1016/j.socscimed.2007.02.007 17374552

[CIT0064] Miller, R. R., Zhang, Y., Silliman, R. A., Hayes, M. K., leveille, S. G., Murabito, J. M., Kiel, D., O’connor, G. T., Felson, D. T. (2004). Effect of medical conditions on improvement in self‐reported and observed functional performance of elders. Journal of the American Geriatrics Society, 52(2), 217–223. 10.1046/j.0002-8614.2004.52057.x 14728630

[CIT0065] Mirecki, R. M., Chou, J. L., Elliott, M., & Schneider, C. M. (2013). What factors influence marital satisfaction? Differences between first and second marriages. Journal of Divorce & Remarriage, 54(1), 78–93. 10.1080/10502556.2012.743831

[CIT0066] Mitra, S., & Sambamoorthi, U. (2014). Disability prevalence among adults: Estimates for 54 countries and progress toward a global estimate. Disability and Rehabilitation, 36(11), 940–947. 10.3109/09638288.2013.825333 23962193

[CIT0067] Moscoe, E., Bor, J., & Bärnighausen, T. (2015). Regression discontinuity designs are underutilized in medicine, epidemiology, and public health: A review of current and best practice. Journal of Clinical Epidemiology, 68(2), 122–133. 10.1016/j.jclinepi.2014.06.021 25579639

[CIT0068] Müller, T., & Shaikh, M. (2018). Your retirement and my health behavior: Evidence on retirement externalities from a fuzzy regression discontinuity design. Journal of Health Economics, 57, 45–59. 10.1016/j.jhealeco.2017.10.005 29182934

[CIT0069] Na, L., & Streim, J. E. (2017). Psychosocial well-being associated with activity of daily living stages among community-dwelling older adults. Gerontology and Geriatric Medicine, 2333, 721417700011. 10.1177/2333721417700011 PMC5433668 28540343

[CIT0070] Neppl, T. K., Senia, J. M., & Donnellan, M. B. (2016). Effects of economic hardship: Testing the family stress model over time. Journal of Family Psychology, 30(1), 12–21. 10.1037/fam0000168 26551658 PMC4742411

[CIT0071] Newmyer, L., Lowrey, K. L., & Levchenko, Y. (2023). Unplanned costs and benefits: Gender and spousal spillover effects of retirement on health. Journal of Marriage and the Family, 85(5), 1110–1124. 10.1111/jomf.12925 38250186 PMC10798816

[CIT0072] Noël-Miller, C. (2010). Longitudinal changes in disabled husbands’ and wives’ receipt of care. Gerontologist, 50(5), 681–693. 10.1093/geront/gnq028 20382664 PMC2937250

[CIT0073] O’Keeffe, A. G., & Baio, G. (2016). Approaches to the estimation of the local average treatment effect in a regression discontinuity design. Scandinavian Journal of Statistics Theory and Applications, 43(4), 978–995. 10.1111/sjos.12224 27867250 PMC5111792

[CIT0074] Okamoto, S., & Kobayashi, E. (2022). The retirement-health puzzle: Breathe a sigh of relief at retirement? *medRxiv*, 2022.2003.2013.22271992. 10.1101/2022.03.13.22271992 36044284

[CIT0075] Ortiz, C., López-Cuadrado, T., Rodríguez-Blázquez, C., Pastor-Barriuso, R., & Galán, I. (2022). Clustering of unhealthy lifestyle behaviors, self-rated health and disability. Preventive Medicine, 155(11), 1069. 10.1016/j.ypmed.2021.106911 34922996

[CIT0076] Park, J. H., Moon, J. H., Kim, H. J., Kong, M. H., & Oh, Y. H. (2020). Sedentary lifestyle: Overview of updated evidence of potential health risks. Korean Journal of Family Medicine, 41(6), 365–373. 10.4082/kjfm.20.0165 33242381 PMC7700832

[CIT0077] Pinsky, J. L., Branch, L. G., Jette, A. M., Haynes, S. G., Feinleib, M., Cornoni-Huntley, J. C., & Bailey, K. R. (1985). Framingham Disability Study: Relationship of disability to cardiovascular risk factors among persons free of diagnosed cardiovascular disease. American Journal of Epidemiology, 122(4), 644–656. 10.1093/oxfordjournals.aje.a114144 3161323

[CIT0078] Pinsky, J. L., Leaverton, P. E., & Stokes III, J. (1987). Predictors of good function: The Framingham Study. Journal of Chronic Diseases, 40, 159S–167S. 10.1016/S0021-9681(87)80045-0 2954993

[CIT0079] Reed, D. M., Foley, D. J., White, L. R., Heimovitz, H., Burchfiel, C. M., & Masaki, K. (1998). Predictors of healthy aging in men with high life expectancies. American Journal of Public Health, 88(10), 1463–1468. 10.2105/ajph.88.10.1463 9772845 PMC1508464

[CIT0080] Schone, B. S., & Weinick, R. M. (1998). Health-related behaviors and the benefits of marriage for elderly persons. Gerontologist, 38(5), 618–627. 10.1093/geront/38.5.618 9803650

[CIT0081] Schünemann, J., Strulik, H., & Trimborn, T. (2017). The gender gap in mortality: How much is explained by behavior? Journal of Health Economics, 54, 79–90. 10.1016/j.jhealeco.2017.04.002 28478344

[CIT0082] Scuteri, A., Spazzafumo, L., Cipriani, L., Gianni, W., Corsonello, A., Cravello, L., Repetto, L., Bustacchini, S., Lattanzio, F., & Sebastiani, M. (2011). Depression, hypertension, and comorbidity: Disentangling their specific effect on disability and cognitive impairment in older subjects. Archives of Gerontology and Geriatrics, 52(3), 253–257. 10.1016/j.archger.2010.04.002 20416961

[CIT0083] Szinovacz, M. E. (2000). Changes in housework after retirement: A panel analysis. Journal of Marriage and Family, 62(1), 78–92. 10.1111/j.1741-3737.2000.00078.x

[CIT0085] Umberson, D. (1987). Family status and health behaviors: Social control as a dimension of social integration. Journal of Health and Social Behavior, 28(3), 306–319. 10.2307/2136848 3680922

[CIT0086] Umberson, D. (1992). Gender, marital status and the social control of health behavior. Social Science & Medicine (1982), 34(8), 907–917. 10.1016/0277-9536(92)90259-s 1604380

[CIT0087] Van Solinge, H. (2007). Health change in retirement: A longitudinal study among older workers in the Netherlands. Research on Aging, 29(3), 225–256. 10.1177/0164027506298223

[CIT0088] Vaughan, L., Leng, X., La Monte, M. J., Tindle, H. A., Cochrane, B. B., & Shumaker, S. A. (2016). Functional independence in late-life: Maintaining physical functioning in older adulthood predicts daily life function after age 80. Journals of Gerontology, Series A: Biological Sciences and Medical Sciences, 71(Suppl 1), S79–86. 10.1093/gerona/glv061 26858328 PMC5865534

[CIT0089] Verbrugge, L. M., & Jette, A. M. (1994). The disablement process. Social Science & Medicine (1982), 38(1), 1–14. 10.1016/0277-9536(94)90294-1 8146699

[CIT0090] Wang, L., Van Belle, G., Kukull, W. B., & Larson, E. B. (2002). Predictors of functional change: A longitudinal study of nondemented people aged 65 and older. Journal of the American Geriatrics Society, 50(9), 1525–1534. 10.1046/j.1532-5415.2002.50408.x 12383150

[CIT0091] Wang, M., & Shultz, K. S. (2010). Employee retirement: A review and recommendations for future investigation. Journal of Management, 36(1), 172–206. 10.1177/0149206309347957

[CIT0092] Yu, X., & Liu, S. (2021). Female labor force status and couple’s marital satisfaction: A Chinese analysis. Frontiers in Psychology, 12, 691460. 10.3389/fpsyg.2021.691460 34367016 PMC8343394

[CIT0093] Zang, E. (2020). Spillover effects of a husband’s retirement on a woman’s health: Evidence from urban China. Social Science & Medicine (1982), 245, 112684. 10.1016/j.socscimed.2019.112684 31765854

[CIT0094] Zhang, C., & Liang, Y. (2023). The impact of education level on marital satisfaction: Evidence from China. Social Sciences & Humanities Open, 7(1), 100487. 10.1016/j.ssaho.2023.100487

[CIT0095] Zhang, Y., Salm, M., & van Soest, A. (2018). The effect of retirement on healthcare utilization: Evidence from China. Journal of Health Economics, 62, 165–177. 10.1016/j.jhealeco.2018.09.009 30390499

[CIT0096] Zhao, Y., Hu, Y., Smith, J. P., Strauss, J., & Yang, G. (2014). Cohort profile: The China Health and Retirement Longitudinal Study (CHARLS). International Journal of Epidemiology, 43(1), 61–68. 10.1093/ije/dys203 23243115 PMC3937970

[CIT0097] Zhong, R., & Ma, A. (2016). The international experience of flexible retirement age and its enlightenment. Journal of Social Sciences, 7(7), 64. https://qikan.cqvip.com/Qikan/Article/Detail?id=669397444

[CIT0098] Zunzunegui, M., Rodriguez-Laso, A., Otero, A., Pluijm, S., Nikula, S., Blumstein, T., Jylhä, M., Minicuci, N., & Deeg, D. (2005). Disability and social ties: Comparative findings of the CLESA study. European Journal of Ageing, 2(1), 40–47. 10.1007/s10433-005-0021-x 28794715 PMC5547668

