# Supplementary material for: The Asymmetric Spillover Effects of Retirement on Disability: Evidence From China
Source: Innov Aging. 2024 Aug 23;8(9):igae074. doi: 10.1093/geroni/igae074 (PMC11441369; doi:10.1093/geroni/igae074)
Supplement: igae074_suppl_Supplementary_Material [file igae074_suppl_supplementary_material.docx]

***Innovation in Aging* Supplementary Material: Bai et al. The Asymmetric Spillover Effects of Retirement on Disability: Evidence From China.**

**Section 1: Supplementary Methods**

RDD requires that there is no manipulation of the assignment variable (Moscoe et al., 2015). Our RDD estimations would be biased if participants had reported false ages to manipulate their eligibility status for retirement. Using a visual assessment of a histogram of age around the retirement threshold (**Supplementary Figure 2**), we found no evidence of such manipulation. The validity of an RDD requires that there are no changes in the values of confounding factors that correlate with the threshold or the outcomes. Therefore, we plotted participants’ characteristics against the assignment variable in **Supplementary Figure 3** and did not observe discontinuity in these characteristics around the threshold. Finally, we formally tested whether there were systematic differences in individual characteristics (i.e., age, middle school completion, and age over 60 years) that were correlated with the threshold by using observed covariates as outcomes and spousal age as an assignment variable. The results are shown in **Supplementary Table 2** and indicate that there is no evidence of systematic differences.

**Section 2: Variables Used in Supplementary Analyses**

The variables used in the supplementary analyses are defined as follows:

1. Physical activity: a constructed variable of a total physical activity score based on the International Physical Activity Questionnaire (IPAQ), which asks about physical activities undertaken for exercise, entertainment, work, and other purposes. General questions about physical activity in the CHARLS questionnaire asked respondents about the amount of time they spent on different types of activities (vigorous activities, moderate activities, and walking for at least 10 minutes continuously) in a usual week. We indexed each respondent’s amount of physical activity in one day as 1 (<0.5 h), 2 (0.5–2 h), 3 (2–4 h), or 4 (>4 h) according to their responses. The weekly PA duration score was calculated by multiplying the number of days and the daily PA duration index for each type of activity. Finally, we generated the variable of PA score using metabolic equivalent (MET) multipliers as follows: PA score = 8.0 × total vigorous activity weekly duration score + 4.0 × total moderate activity weekly duration score + 3.3 × total walking weekly duration score.
2. Chronic diseases: defined as a dichotomous variable with an assigned value of one if a respondent had any of the following diseases: hypertension, dyslipidemia, diabetes, malignant tumor, chronic lung disease, liver disease, heart disease, stroke, kidney disease, digestive disease, psychiatric problems, memory-related disease, arthritis or rheumatism, or asthma.
3. Kidney disease: defined as a dichotomous variable with an assigned value of one if a respondent had kidney disease (except for tumors or cancer) and zero otherwise.
4. Asthma: defined as a dichotomous variable with an assigned value of one if a respondent had asthma and zero otherwise.
5. Liver disease: defined as a dichotomous variable with an assigned value of one if a respondent had liver disease (except for fatty liver, tumors, or cancer) and zero otherwise.
6. Digestive disease: defined as a dichotomous variable with an assigned value of one if a respondent had a stomach or other digestive disease (except for tumors or cancer) and zero otherwise.

**Section 3: Supplementary Data Elements**

**Supplementary Figure 1:** Participant Selection Diagram

**
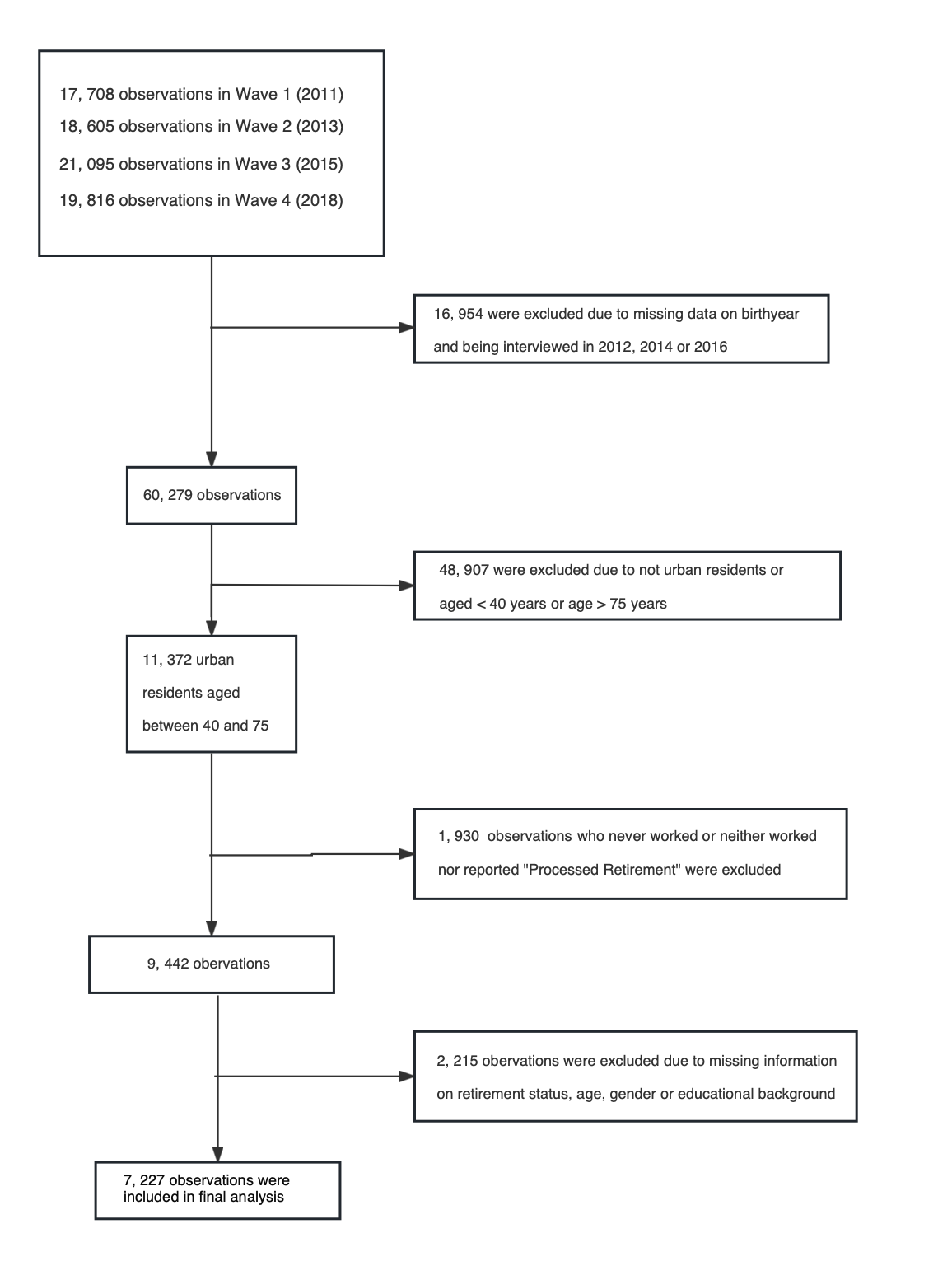
**

**Supplementary Figure 2:** Manipulation test for running variable: Distribution of normalized ages among wives and husbands


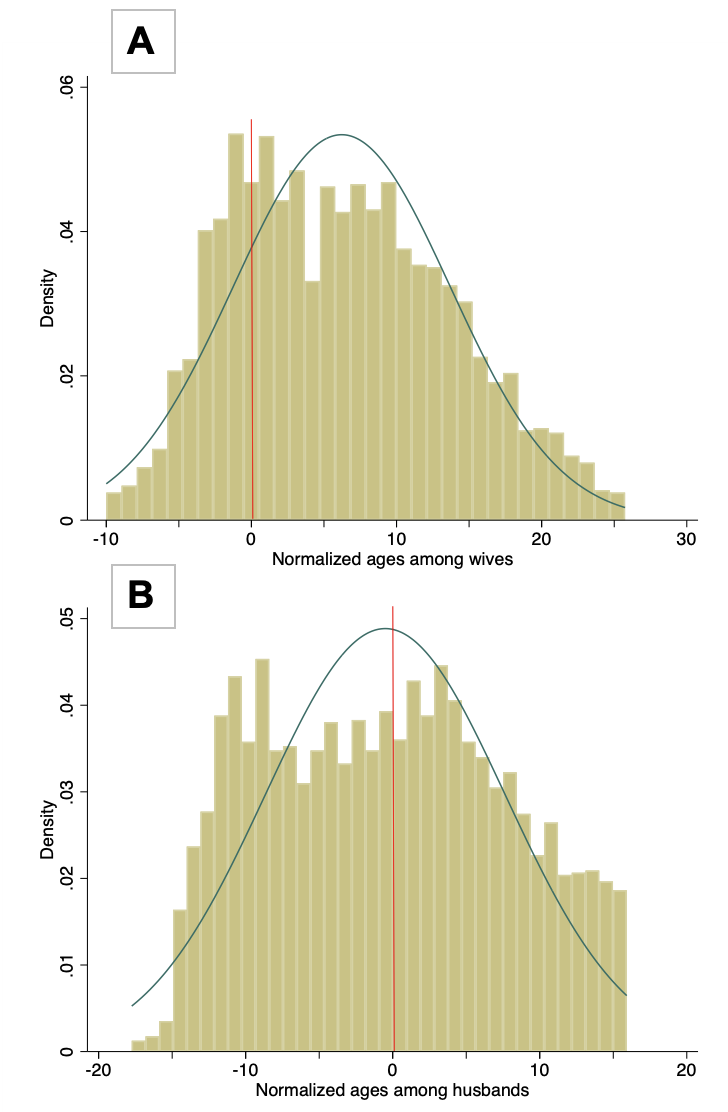


Note: The x-axis is spousal age relative to the mandatory retirement age. (A) represents the manipulation test for the running variable among wives. (B) represents the manipulation test for the running variable among husbands.

**Supplementary Figure 3:** Manipulation test for running variable: RDD plots of covariates

Note: (A) represents RDD plots of husbands’ predetermined characteristics and wives’ ages, with 95% confidence intervals and a linear fit. (B) represents RDD plots of wives’ predetermined characteristics and husbands’ ages, with 95% confidence intervals and a linear fit. The point estimates presented here are based on a data-driven method with bandwidths that are much smaller than the range displayed in the figure.

**Supplementary Figure 4:** RDD plots of spouses’ physical health

Note: (A) represents RDD plots of husbands’ physical health and wives’ ages, with 95% confidence intervals and a linear fit. (B) represents RDD plots of wives’ physical health and husbands’ ages, with 95% confidence intervals and a linear fit. The point estimates presented here are based on a data-driven method with bandwidths that are much smaller than the range displayed in the figure.

**Supplementary Table 1**: Comparison of the Sample Dropped and the Sample Used

| Variables | Sample Used  (N=7227) |  | Sample Dropped  (N=4145) | *p* value |
| --- | --- | --- | --- | --- |
|  | **Mean (SD)** | **N** | **Mean (SD)** |  |
| **Outcomes** |  |  |  |  |
| ADL score | 6.47 (1.54) | 2589 | 6.67 (2.06) | 0.085 |
| IADL score | 5.48 (1.76) | 1914 | 5.69 (2.28) | 0.021 |
| **Covariates** |  |  |  |  |
| Age | 57.17 (7.87) | 4145 | 59.07 (8.24) | <.001 |
| Spouse’s age | 57.67 (8.05) | 4145 | 58.97 (8.29) | <.001 |
| Middle school graduate or higher | 0.67 (0.47) | 3904 | 0.62 (0.49) | <.001 |
| Spouse middle school graduate or higher | 0.73 (0.44) | 3971 | 0.63 (0.48) | <.001 |

**Supplementary Table 2**: Falsification test

|  | Husband’s middle school completion | Husband’ age | Husband’s age over 60 |
| --- | --- | --- | --- |
| Conventional | 0.056 | 2.130 | -0.008 |
|  | (0.248) | (1.526) | (0.039) |
| Robust | 0.132 | 2.371 | 0.004 |
|  | (0.284) | (1.751) | (0.050) |
| Bandwidth | 3.828 | 3.490 | 3.034 |
| N | 2,983 | 2,983 | 2,983 |
|  | Wife’s middle school completion | Wife’s age | Wife’s age over 60 |
| Conventional | 0.032 | -0.360 | -0.008 |
|  | (0.292) | (1.700) | (0.039) |
| Robust | 0.095 | -0.799 | 0.004 |
|  | (0.336) | (2.083) | (0.050) |
| Bandwidth | 4.001 | 4.145 | 3.034 |
| N | 4,244 | 4,244 | 2,983 |

Notes: The sample is from the 2011, 2013, 2015, and 2018 waves of CHARLS. Covariate-adjusted local linear fuzzy RDD estimation with (robust) and without (conventional) bias-adjustment of point estimation and inference. All standard errors (in parentheses) are clustered with plug-in residuals at the individual level. No covariates are included. Bandwidth (common on both sides of the threshold) is selected based on MSE-optimal bandwidth selector (Cattaneo et al., 2019). *** p<0.01, ** p<0.05, * p<0.1.

**Supplementary Table 3:** The effects of wife’s retirement on her husband’s disability: different bandwidths

| Bandwidth rescaling factor |  | 50% | 75% | 125% | 150% |
| --- | --- | --- | --- | --- | --- |
| ADL score | Conventional | -0.639** | -0.676*** | -0.555*** | -0.342* |
|  |  | (0.252) | (0.251) | (0.178) | (0.190) |
|  | Bias-corrected | -0.643** | -0.524** | -0.702*** | -0.598*** |
|  |  | (0.252) | (0.251) | (0.178) | (0.190) |
|  | Robust | -0.643** | -0.524* | -0.702** | -0.598** |
|  |  | (0.272) | (0.315) | (0.277) | (0.277) |
|  | Effective Number of observation | 154 | 219 | 354 | 415 |
| IADL score | Conventional | -0.856*** | -0.743*** | -0.620*** | -0.535*** |
|  |  | (0.301) | (0.282) | (0.211) | (0.207) |
|  | Bias-corrected | -0.620** | -0.782*** | -0.766*** | -0.682*** |
|  |  | (0.301) | (0.282) | (0.211) | (0.207) |
|  | Robust | -0.620 | -0.782** | -0.766*** | -0.682*** |
|  |  | (0.398) | (0.335) | (0.274) | (0.262) |
|  | Effective Number of observation | 387 | 518 | 840 | 961 |

Notes: The sample is from the 2011, 2013, 2015, and 2018 waves of CHARLS. Covariate-adjusted local linear fuzzy RDD estimation with (robust) and without (conventional) bias-adjustment of point estimation and inference. All standard errors (in parentheses) are clustered with plug-in residuals at the individual level. Covariates include husband’s age and age squared; wife’s age, age squared, and age cubed; whether the husband is older than 60 years; husbands’ middle school completion status; husbands’ childhood health; and year dummies. *** p<0.01, ** p<0.05, * p<0.1.

**Supplementary Table 4:** The effects of wife’s retirement on her husband’s disability: donut hole estimation

| Size of donut hole (each side) | | 0 months | 1 month | 2 months | 3 months |
| --- | --- | --- | --- | --- | --- |
| ADL score | Conventional | -0.924*** | -0.560*** | -0.997*** | -0.972*** |
|  |  | (0.190) | (0.181) | (0.306) | (0.334) |
|  | Robust | -0.673*** | -0.338 | -0.695 | -0.665 |
|  |  | (0.249) | (0.226) | (0.436) | (0.494) |
|  | Bandwidth | 2.915 | 3.611 | 2.614 | 2.212 |
|  | N | 1,387 | 1,386 | 1,363 | 1,354 |
| IADL score | Conventional | -1.417*** | -1.366*** | -0.703* | -2.635 |
|  |  | (0.500) | (0.494) | (0.363) | (1.809) |
|  | Robust | -0.134 | -0.051 | -1.123** | 4.194* |
|  |  | (0.604) | (0.606) | (0.473) | (2.458) |
|  | Bandwidth | 2.698 | 2.711 | 2.022 | 2.263 |
|  | N | 2,970 | 2,964 | 2,906 | 2,878 |

Notes: The sample is from the 2011, 2013, 2015, and 2018 waves of CHARLS. Covariate-adjusted local linear fuzzy RDD estimation with (robust) and without (conventional) bias-adjustment of point estimation and inference. All standard errors (in parentheses) are clustered with plug-in residuals at the individual level. Covariates include wife’s age and age squared; husband’s age, age squared, and age cubed; whether the wife is older than 60 years; husband and wife’s middle school completion status; and year dummies. Bandwidth (common on both sides of the threshold) is selected based on MSE-optimal bandwidth selector (Cattaneo et al., 2019). *** p<0.01, ** p<0.05, * p<0.1.

**Supplementary Table 5:** Effect of a wife’s retirement on her husband’s health behaviors, emotions, and disease diagnoses

| Behaviors | Current Smoker | Excessive Drinker | Physically Active | Social Participation |  |
| --- | --- | --- | --- | --- | --- |
| Conventional | -0.191 | -0.988* | -0.727** | 0.591 |  |
|  | (0.260) | (0.539) | (0.283) | (0.459) |  |
| Robust | -0.115 | -0.430 | -0.403 | 0.377 |  |
|  | (0.313) | (0.687) | (0.359) | (0.566) |  |
| Bandwidth | 2.696 | 2.667 | 2.762 | 2.478 |  |
| N | 2,851 | 5,067 | 4,112 | 5,066 |  |
| Emotions | Depressed days | Happy days |  |  |  |
| Conventional | -3.582*** | 4.049*** |  |  |  |
|  | (1.299) | (1.252) |  |  |  |
| Robust | -3.322** | 3.235** |  |  |  |
|  | (1.470) | (1.444) |  |  |  |
| Bandwidth | 2.486 | 2.589 |  |  |  |
| N | 4,451 | 4,441 |  |  |  |
| Disease diagnoses | Chronic diseases | Kidney disease | Asthma | Liver disease | Digestive disease |
| Conventional | 0.612 | 1.233* | 0.091 | 0.295 | 0.076 |
|  | (0.638) | (0.669) | (0.068) | (0.294) | (0.330) |
| Robust | 0.593 | 0.746 | 0.032 | 0.053 | 0.259 |
|  | (0.786) | (0.857) | (0.079) | (0.373) | (0.393) |
| Bandwidth | 2.538 | 2.370 | 2.471 | 2.613 | 2.031 |
| N | 5,067 | 4,934 | 4,922 | 4,936 | 4,920 |

Notes: Current smoking status was categorized as current smoking or non-current smoking using the question “Do you smoke cigarettes now?” (yes or no); Excessive alcohol use was defined as more than 14 drinks per week for men and more than seven drinks per week for women following the National Institute on Alcohol Abuse and Alcoholism guidelines. Alcohol consumption was measured by multiplying the number of days per week that alcohol was consumed by the number of drinks per day, which resulted in the number of drinks per week. Participants that were not in this alcohol consumption range were classified as non-excessive alcohol use; Physical activity (PA) was constructed based on the International Physical Activity Questionnaire (IPAQ), and participants with PA score above highest tertile was defined as physically active (a dichotomous PA variable as 1), and PA score below highest tertile was defined as physically inactive (a dichotomous PA variable as 0); Social participation is assigned a value of 1 if the respondent participated in any of 11 specified social activities in the past month and 0 otherwise; Happy days: the number of self-reported happy days experienced in the past week; Depressed days: the number of self-reported depressed days experienced in the past week; Chronic diseases/Kidney disease/Asthma/Liver disease/Digestive disease: each variable was assigned a value of 1 if the respondent was diagnosed with the given disease by a doctor.
